# Biogas Conversion to Syngas Using Advanced Ni-Promoted Pyrochlore Catalysts: Effect of the CH_4_/CO_2_ Ratio

**DOI:** 10.3389/fchem.2021.672419

**Published:** 2021-04-14

**Authors:** Estelle le Saché, Andrea Alvarez Moreno, Tomas Ramirez Reina

**Affiliations:** ^1^Department of Chemical and Process Engineering, University of Surrey, Guildford, United Kingdom; ^2^Estado Sólido y Catálisis Ambiental, Departamento de Química, Facultad de Ciencias, Universidad Nacional de Colombia, Bogotá, Colombia; ^3^Inorganic Chemistry Department, Material Science Institute, University of Seville-CSIC, Seville, Spain

**Keywords:** biogas, dry reforming, Ni catalysts, CH_4_/CO_2_ ratio, bioenergy

## Abstract

Biogas is defined as the mixture of CH_4_ and CO_2_ produced by the anaerobic digestion of biomass. This particular mixture can be transformed in high valuable intermediates such as syngas through a process known as dry reforming (DRM). The reaction involved is highly endothermic, and catalysts capable to endure carbon deposition and metal particle sintering are required. Ni-pyrochlore catalysts have shown outstanding results in the DRM. However, most reported data deals with CH_4_/CO_2_ stoichiometric ratios resulting is a very narrow picture of the overall biogas upgrading via DRM. Therefore, this study explores the performance of an optimized Ni-doped pyrochlore, and Ni-impregnated pyrochlore catalysts in the dry reforming of methane, under different CH_4_/CO_2_ ratios, in order to simulate various representatives waste biomass feedstocks. Long-term stability tests showed that the ratio CH_4_/CO_2_ in the feed gas stream has an important influence in the catalysts' deactivation. Ni doped pyrochlore catalyst, presents less deactivation than the Ni-impregnated pyrochlore. However, biogas mixtures with a CH_4_ content higher than 60%, lead to a stronger deactivation in both Ni-catalysts. These results were in agreement with the thermogravimetric analysis (TGA) of the post reacted samples that showed a very limited carbon formation when using biogas mixtures with CH_4_ content <60%, but CH_4_/CO_2_ ratios higher than 1.25 lead to an evident carbon deposition. TGA analysis of the post reacted Ni impregnated pyrochlore, showed the highest amount of carbon deposited, even with lower stoichiometric CH_4_/CO_2_ ratios. The later result indicates that stabilization of Ni in the pyrochlore structure is vital, in order to enhance the coke resistance of this type of catalysts.

## Introduction

In the continuous search of renewable energy sources, the world has turned again toward the use of biomass. Biomass represents an alternative to fossil fuels and could be as versatile through several conversion processes including combustion, pyrolysis, fermentation, gasification, and anaerobic digestion (Papadopoulou et al., 2012).

While the combustion of biomass allows the production of heat and electricity, pyrolysis, and fermentation processes allow the production of liquid fuels suitable for combustion engines or liquid energy carriers. Thermal biomass gasification produces a gas mixture composed of H_2_, CO, CH_4_, CO_2_, H_2_O, N_2_, and light hydrocarbons. However, this process is energy intensive due to the high moisture content of biomass, and require critical process demands due to the large production of tar (Neubauer, 2013; Ren et al., 2020). Anaerobic digestion on the other hand, is based on the decomposition of organic matter in the absence of O_2_ by bacterial action and requires much less energy than gasification or combustion. Through this process, several types of biomass can be converted into a gas mixture of methane (CH_4_) and carbon dioxide (CO_2_), known as biogas (Wu et al., 2019).

This particular gas mixture can be considered quite contaminant, since both molecules are well-recognized greenhouse gases, however, when coupling this mixture with the CO_2_ reforming process (also known as dry reforming), high valuable intermediates such as synthesis gas (syngas) can be generated (equation 1).

(1) $$\begin{array}{r} \text{C}{\text{H}}_{4}+\text{C}{\text{O}}_{2}⇆2\;{\text{H}}_{2}+2\text{ CO }\Delta {\text{H}}_{298}=+247\text{ kJ mo}{\text{l}}^{-1} \end{array}$$

Syngas is known to be a primary feedstock to synthesize fuels and chemicals such as methanol, DME, as well as long-chained hydrocarbons via the Fischer-Tropsch process (Santos and Alencar, 2020; Zhao et al., 2020). Additionally, it has been suggested as a feed gas to high temperature solid oxide fuel cells (SOFCs) for electricity generation (Lanzini and Leone, 2010; Shiratori et al., 2010).

CO_2_ reforming has been reported as one of the main strategies in the carbon capture and utilization (CCU) approach. Still, from a CO_2_ emissions mitigation perspective, dry reforming (DRM) has a limited impact considering the high energy input required to reach operating conditions. However, when coupling DRM with biomass utilization, its environmental advantage is incredibly boosted.

Municipal waste, sewage sludge, animal manure, and agricultural waste are widely known to be the characteristic feedstocks for biogas production. Nevertheless, the biogas composition may vary upon the biodegradable source since it is dependent on the composition, density and water content of the source. While landfill waste can generate a biogas with a CH_4_ content around 40% and a CO_2_ content around 40%, agricultural waste can generate a biogas containing 70% of CH_4_, and a 30% of CO_2_. Representative feedstocks and their corresponding biogas compositions are listed in Table 1.

**Table 1:** Biogas from different feedstocks.

**Biogas source**	**CH_**4**_ (%)**	**CO_**2**_ (%)**	**N_**2**_ (%)**
Landfill Waste	45–62	24–40	1–17
Sewage Waste	58–65	33–40	1–8
Organic Waste	60–70	30–40	1–5

*Data obtained from Papadopoulou et al. (2012) and Ullah Khan et al. (2017)*.

Syngas generation through dry reforming of biogas is usually performed with metal-based catalysts. However, some critical characteristics are required in these solids. To begin with, they need to be able to activate both molecules (CH_4_ and CO_2_). Then, a strong resistance toward coke deposition is required, as the reaction is extremely endothermic and methane decomposition is favored on the same temperature range. Lastly, they must be stable in order to avoid sintering of the metal particles and subsequent loss of active surface. Noble metal catalysts (based in Rh, Ru, Pd) fulfill all of these requirements (Abdulrasheed et al., 2019; Aziz et al., 2019), however, for large-scale applications, low-cost transition metals are preferred. Among them, Ni-based catalysts standout. They are much cheaper and have comparable catalytic activities with noble metals. Nevertheless, coke deposition and sintering of Ni particles, are their most important drawbacks. In order to overcome this challenge, strategies regarding the stabilization of Ni, have been proposed. The use of supports such as fluorites, hexaaluminates, perovskites, and pyrochlores, has been investigated for this purpose (Dama et al., 2018; le Saché et al., 2018, 2020).

Pyrochlores are a very interesting family of materials. Generally speaking, they are defined as mixed oxides with the general formula A_2_B_2_O_7_. “A” represents a large trivalent cation, typically a rare-earth metal such as La, and “B” represents a tetravalent cation of smaller diameter, typically a transition metal such as Zr (Shukla et al., 2015). Pyrochlores present a high thermal stability and high oxygen mobility which makes them excellent candidates for high temperature and coke resistance operations (Zhang et al., 2017). Previous work in our group showed that the substitution of 10 wt.% Ni on the B site of a La_2_Zr_2_O_7_ pyrochlore led to a very active, stable, and carbon resistant catalyst for dry reforming of methane (DRM) (le Saché et al., 2018; Reina et al., 2020). Likewise, the Ni impregnated pyrochlore structure has also been reported as active in the DRM (le Saché et al., 2020). However, these catalysts have only been tested under CH_4_/CO_2_ stoichiometric ratios. Therefore, this work studies the performance of the optimized Ni-doped pyrochlore, and compares it with Ni-impregnated pyrochlore catalysts in the DRM under different CH_4_/CO_2_ ratios, in order to simulate different waste biomass feedstocks. The work aims to showcase suitable upgrading routes for different types of biogas by the CO_2_ reforming process.

## Materials and Methods

### Catalyst Preparation

The 10% Ni-doped pyrochlore was synthesized using a modified citrate method described elsewhere (le Saché et al., 2018, 2020; Reina et al., 2020). In summary, lanthanum nitrate [La(NO_3_)_3_·6H_2_O], nickel nitrate [Ni(NO_3_)_2_·6H_2_O], and zirconyl nitrate [ZrO(NO_3_)_2_·6H_2_O] were used as precursors. Salts were dissolved in deionized, and then mixed with a citric acid solution. The required amount of each precursor was adjusted to reach a Ni loading of 10 wt.%. The solution was concentrated in a rotary evaporator and then dried for 12 h at 100°C to then be combusted at 200°C. Finally, all solids were calcined at 1,000°C for 8 h. Ni-doped pyrochlore will be referred as LNZ10.

A second catalyst was prepared by the wet impregnation of Ni on the un-doped pyrochlore. The La_2_Zr_2_O_7_ support was prepared by the same citrate method. Ni was then impregnated to reach a Ni loading of 10 wt.%. A 1 M solution of Ni(NO_3_)_2_·6H_2_O (Sigma Aldrich, 99.999%) in ethanol was added to the support and stirred for 2 h. The solvent was removed in a rotary evaporator and the resulting powder was dried for 12 h at 100°C before calcination at 500°C for 4 h. The impregnated catalyst will be referred as Ni/LZ.

### Thermodynamic Analysis

The thermodynamic equilibrium calculations over a range of temperatures, was performed by the Gibbs free energy minimization method using the ChemCad 6.5.5 software. Soave-Redlich-Kwong equation of state was used for fugacity calculations due to its large range applicability in terms of temperature (Perry et al., 1997).

### Catalytic Behavior

The reforming of various biogas mixtures was performed in a down flow fixed bed tubular quartz reactor placed in a tubular furnace. The temperature of the catalytic bed is monitored by a K-type thermocouple and recorded using PicoLog 5 software. The gaseous reactants are mixed in a mixing chamber and fed into the reactor by mass flow controllers from Aalborg. The gas stream may be redirected to a bubble flow meter by means of a 3-valve. At the reactor outlet, the product stream passes through two coalescence filters from Headline Filters to separate any liquid product. The remaining gas flow is then sent to an online gas chromatograph HP 6890 from Agilent equipped with a Carboxen-1000 packed column and a TCD detector.

The reforming of biogas was performed on 100 mg of catalyst at a WHSV of 30 L g^−1^ h^−1^. Prior to reaction, the catalyst was reduced for 1 h at 700°C in 10 vol.% H_2_/N_2_. Temperature screening experiments were performed for temperatures ranging from 550 to 850°C in 50°C increments of 45 min. Time dependent experiments were performed at 700°C for 24 h. The catalytic performance was measured for CH_4_/CO_2_ molar ratios of 1, 1.25, 1.5, and 1.85 balanced in N_2_ in order to model biogas mixtures produced from a range of residues (Table 2).

**Table 2:** Biogas mixtures used in the catalytic testing.

**Biogas mixture**	**CH_**4**_ (%)**	**CO_**2**_ (%)**	**CH_**4**_/CO_**2**_ molar ratio**
Stoichiometric mixture	50	50	1
1	55	45	1.25
2	60	40	1.5
3	65	35	1.85

### Catalysts Characterization

#### CH_4_-Temperature-Programmed Surface Reaction

CH_4_-TPSR took place in a locally built fixed bed quartz reactor coupled with a Pfeiffer Vacuum OmniStar GSD 320 mass spectrometer. The experiment was performed on the catalysts reduced *in-situ* at 700°C for 1 h. The temperature was risen to 900°C at a heating rate of 10°C min^−1^ and maintained for 30 min. A flow of 10 vol.% CH_4_/Ar was passed through the reactor and mass to charge ratio (m/z) of 2, 15, 18, 28, and 44 corresponding to H_2_, CH_4_, H_2_O, CO, and CO_2_, respectively, were recorded.

#### X-Ray Powder Diffraction

XRD analysis was undertaken using a PANalytical X'Pert Powder. The samples were analyzed after calcination, reduction and after reaction. The diffraction patterns were recorded with Cu Kα (30 mA, 40 kV) over a 2θ range of 10–90°, using a position sensitive detector with a step size of 0.05° and a step time of 450 s. The powder XRD patterns were further processed using the software X'PertHighscore Plus©.

#### Transmission Electron Microscopy

TEM images were taken with a JEOL electron microscope (model JEM-2010) working at 200 kV. It was equipped with an INCA Energy TEM 100 analytical system and a SIS MegaView II camera. Samples for analysis were suspended in ethanol and placed on copper grids with a holey-carbon film support.

#### Thermogravimetric Analysis

TGA was performed on the spent catalysts in an SDT Q600 V8.3 from TA Instruments. The sample was ramped from room temperature to 900°C at a rate of 10°C min^−1^. Experiments were conducted in 100 mL min^−1^ of Air.

## Results and Discussion

### Thermodynamic Analysis

**Figure 1:**
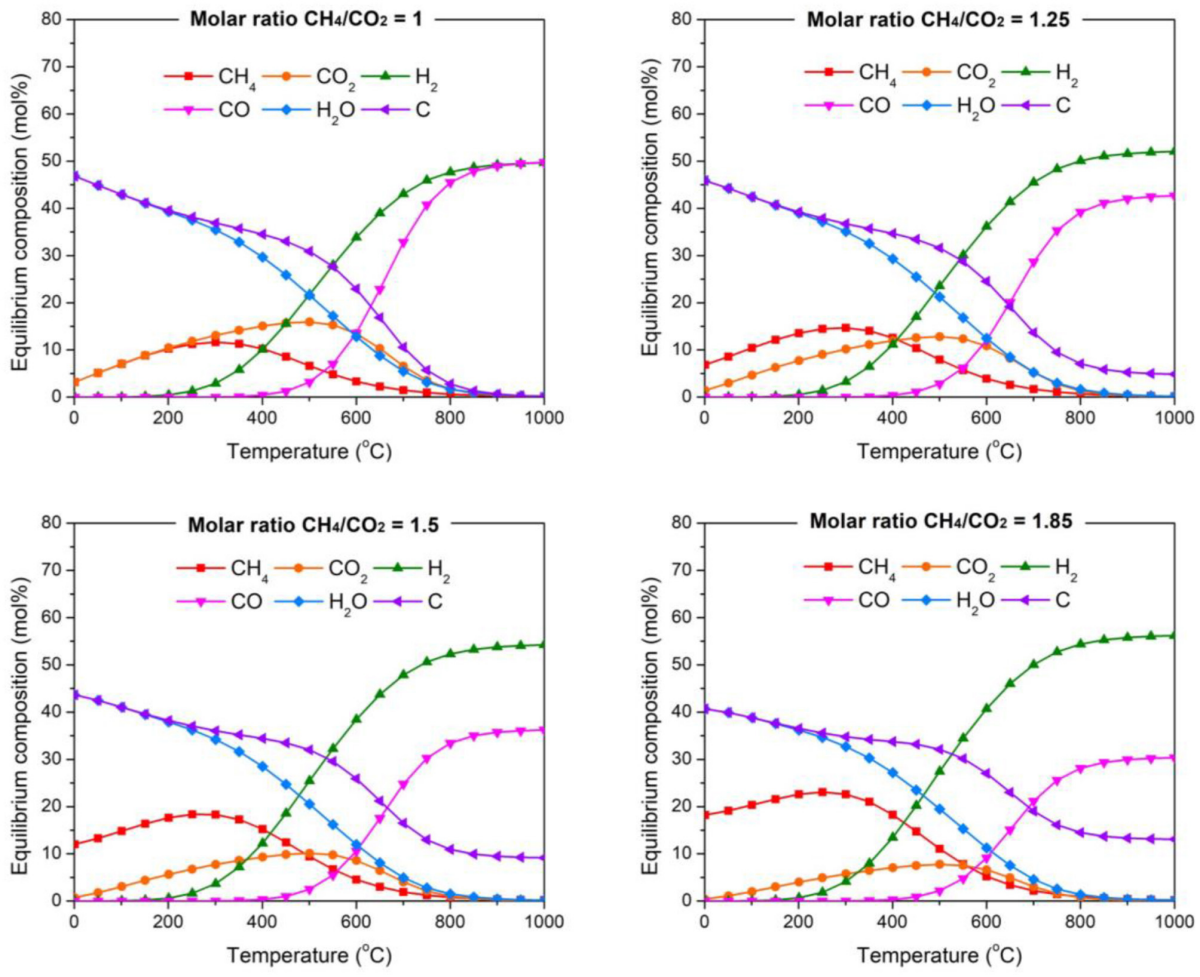
Thermodynamic equilibrium plots of DRM under different biogas mixtures at 1 bar using ChemCad 6.5.5 software.

Figure 1, simulates the DRM reaction under the different CH_4_/CO_2_ ratios described in Table 2. In each graph two sections can be evidenced. First, from 0 to 500°C a higher concentration of carbon and water is observed, with the presence of the main reactants CH_4_ and CO_2_. Secondly, from 500°C onwards the concentrations of CH_4_ and CO_2_ gradually decrease as H_2_ and CO production is evidenced, accompanied with carbon formation.

The low temperature zone is dominated by the Boudouard reaction (equation 2) and the reverse gasification of carbon (equation 3) (Zheng et al., 2017).

(2) $$\begin{array}{r} 2\text{CO }⇆\text{ C}+\text{C}{\text{O}}_{2}\;\Delta {\text{H}}_{298}=-172\text{ kJ mo}{\text{l}}^{-1} \end{array}$$

(3) $$\begin{array}{r} \text{CO}+{\text{H}}_{2}\;⇆\text{ C}+{\text{H}}_{2}\text{O }\Delta {\text{H}}_{298}=-131\text{ kJ mo}{\text{l}}^{-1} \end{array}$$

The presence of CO and water is owed to the reverse water gas shift (RGWS) (equation 4) that is present in a very wide range of temperatures (Chein et al., 2015).

(4) $$\begin{array}{r} \text{C}{\text{O}}_{2}+{\text{H}}_{2}\;⇆\text{ CO}+{\text{H}}_{2}\text{O }\Delta {\text{H}}_{298}=41.4\text{ kJ mo}{\text{l}}^{-1} \end{array}$$

On the other hand, the high temperature area of the graph is characterized by the DRM reaction (equation 1), which agrees with the low CH_4_ and CO_2_ concentration, while H_2_ and CO are the primary products. The carbon present, is mainly owed to the decomposition of methane (equation 5) that is favored at high temperatures (Zhang et al., 2007).

(5) $$\begin{array}{r} \text{C}{\text{H}}_{4}\;⇆\text{ C}+2{\text{H}}_{2}\;\Delta {\text{H}}_{298}=75\text{ kJ mo}{\text{l}}^{-1} \end{array}$$

Since the consumption of CO_2_ occurs at temperatures higher than 500°C, the 550–850°C temperature range was selected for the catalytic tests, where carbon deposition is likely to occur. Regarding the different CH_4_/CO_2_ ratios, it is clearly evidenced that the amount of H_2_ and C in the stream increases with CH_4_/CO_2_ ratio, owing mainly to the excess methane at the inlet stream. In this way, the present simulation also provides information about the syngas composition that could be produced if DRM was performed with various CH_4_/CO_2_ ratios. Increasing the CH_4_/CO_2_ ratio, the H_2_ content can reach up to 55% and the CO content, 35%. This imply that the H_2_/CO ratio could be >1, suggesting that biogas reforming could produce syngas suitable for a wider range of products through the Fischer-Tropsch process.

### CH_4_-Temperature-Programmed Surface Reaction

**Figure 2:**
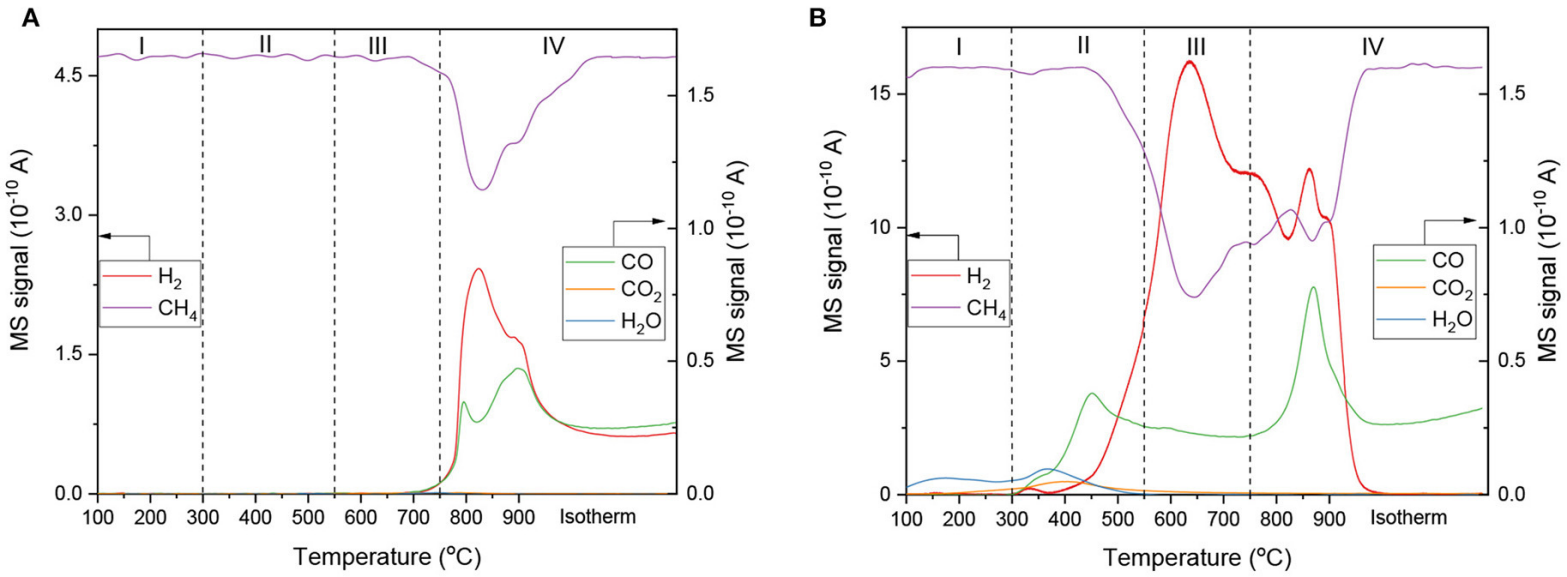
CH_4_-TPSR profiles of **(A)** LZ, and **(B)** LNZ10.

The contribution of the pyrochlore and Ni to activate methane was studied by means of CH_4_-TPSR. In this experiment, solely CH_4_ is introduced in the reactor, which can also be used to monitor the participation of active lattice oxygen during reaction. The absence of O_2_ or CO_2_ in the feed, allows to study the lattice oxygen conductivity in the pyrochlore through the formation of CO or CO_2_ (Pakhare et al., 2012). The support La_2_Zr_2_O_7_ pyrochlore (LZ) was compared to the doped sample LNZ10. The desorption profiles of the reagent and products arising from the reaction are shown in Figure 2. Four temperature regions can be distinguished, corresponding to different dissociation processes. At temperatures lower than 300°C, no dissociation occurs. In the 300–550°C temperature range, a minor methane consumption is detected in the Ni containing catalyst. This process is accompanied by the production of water, carbon monoxide, carbon dioxide, and hydrogen, indicating some methane decomposition on Ni active sites, and methane combustion with adsorbed surface oxygen, following Equation (6) (Khajonvittayakul et al., 2019). Methane combustion was not detected on the bare pyrochlore indicating the lack of easily accessible surface oxygen. On the other hand, this reaction occurred on LNZ10, indicating the higher accessibility of surface oxygen in the doped catalysts. The production of CO at *ca*. 450°C could also be due to the reaction of oxygen associated with Ni in the pyrochlore/perovskite structure as suggested by Pakhare et al. with doped pyrochlores (Pakhare et al., 2012).

(6) $$\begin{array}{r} \text{C}{\text{H}}_{4}+\;4{\text{O}}^{*}\to \text{C}{\text{O}}_{2}+2{\text{H}}_{2}\text{O} \end{array}$$

The activation mechanism occurring between 550 and 750°C is referred as region III in Figure 2. Here, a rapid hydrogen production is witnessed, along a major methane consumption on the Ni doped sample. The limited CO production suggests that this process is mainly due to CH_4_ dissociation on Ni active sites according to Equation (7). This indicates that to avoid carbon deposition, CO_2_ must be readily activated at temperature as low as 500°C.

(7) $$\begin{array}{r} \text{C}{\text{H}}_{4}+\text{ Ni}\to \text{Ni}-\text{C}+2{\text{H}}_{2} \end{array}$$

Lastly, in region IV, at temperatures above 750°C, a final CH_4_ consumption occurs, forming larger amount of CO. This process is observed for both samples, including the bare La_2_Zr_2_O_7_ pyrochlore, implying that oxygen from the pyrochlore lattice is able to dissociate methane at high temperature, following (equation 8) (Zheng et al., 2017).

(8) $$\begin{array}{r} \text{C}{\text{H}}_{4}+\;{\text{O}}^{*}\to \text{CO}+2{\text{H}}_{2} \end{array}$$

Additionally, on the Ni containing catalyst, the coked *Ni-C* sites resulting from Equation (7) can supposedly further react with lattice oxygen to form CO, regenerating *Ni-C* to its original active form Ni. Although methane could still potentially dissociate on *Ni-C* sites, leading to the formation of filaments of carbon, the complete decline of H_2_ production during the high temperature isotherm suggests otherwise.

Overall the doped catalyst was able to dissociate methane at temperatures as low as 400°C, which sets them as good candidates for low temperature dry reforming. Additionally, the pyrochlore structure was found to have a good oxygen storage capacity, with some surface oxygen and active lattice oxygen.

### Catalytic Behavior

#### Temperature Effect

**Figure 3:**
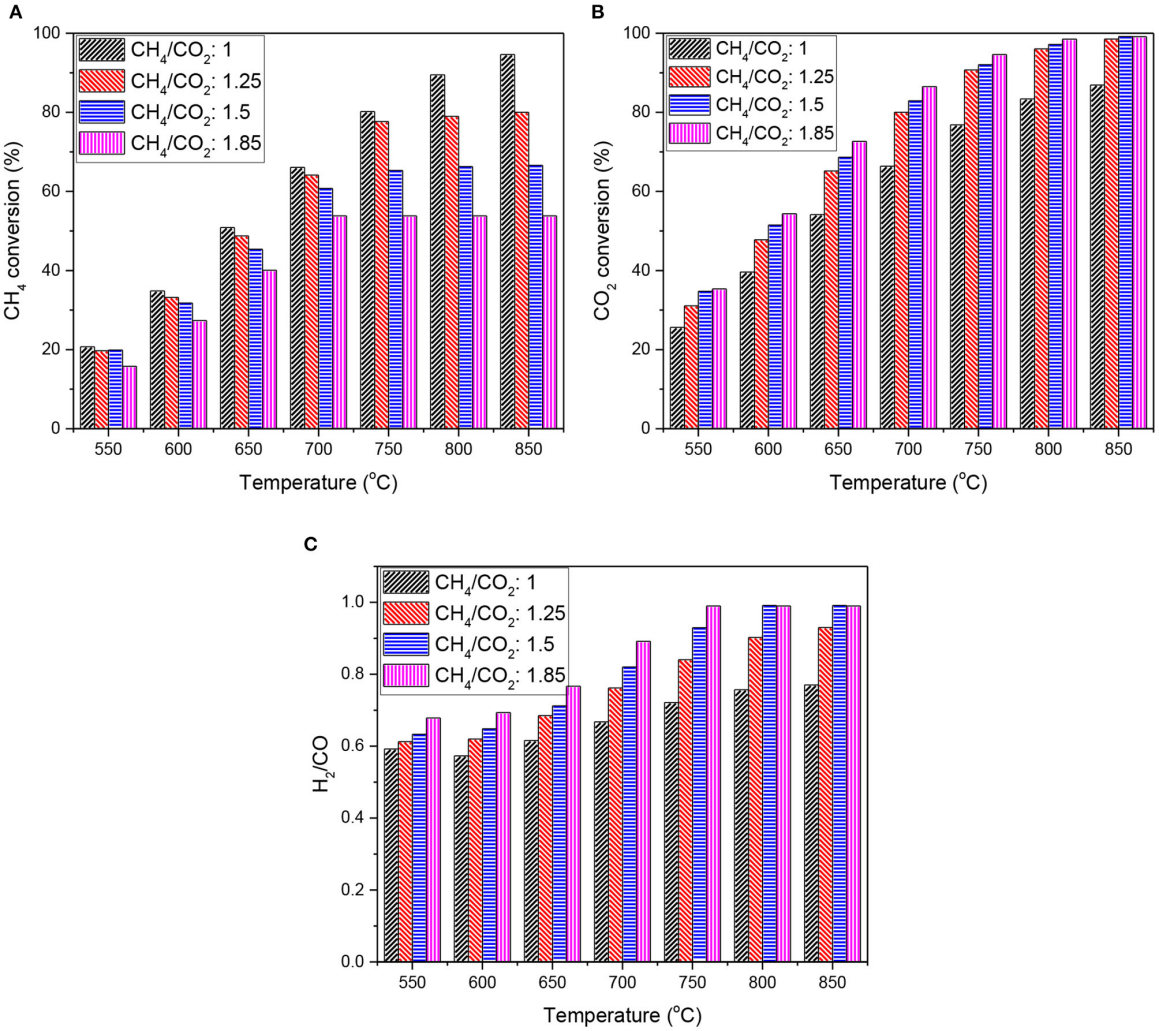
Temperature dependent reaction using different biogas feedstock on LNZ10 **(A)** CH_4_ conversion and **(B)** CO_2_ conversion **(C)** H_2_/CO ratio.

Dry reforming of various biogas mixtures was performed on the doped catalyst, LNZ10. The effect of temperature on CH_4_ and CO_2_ conversions, besides the H_2_/CO ratio obtained, is represented in Figure 3. The catalyst achieved high conversions at relatively low temperatures. Thermodynamic equilibrium conversions were reached at temperature above 700°C. The conversion of methane decreased upon increasing methane content. In particular, methane conversion is limited at high temperature as suggested by thermodynamic calculations. CO_2_ conversions on the other hand, are very similar between biogas feedstock. For CH_4_/CO_2_ superior to 1, CO_2_ is the limiting reactant resulting in high conversions. Additionally, the H_2_/CO ratio produced increases with methane concentration in the feed stream as suggested thermodynamically.

#### Time Dependent Performance

**Figure 4:**
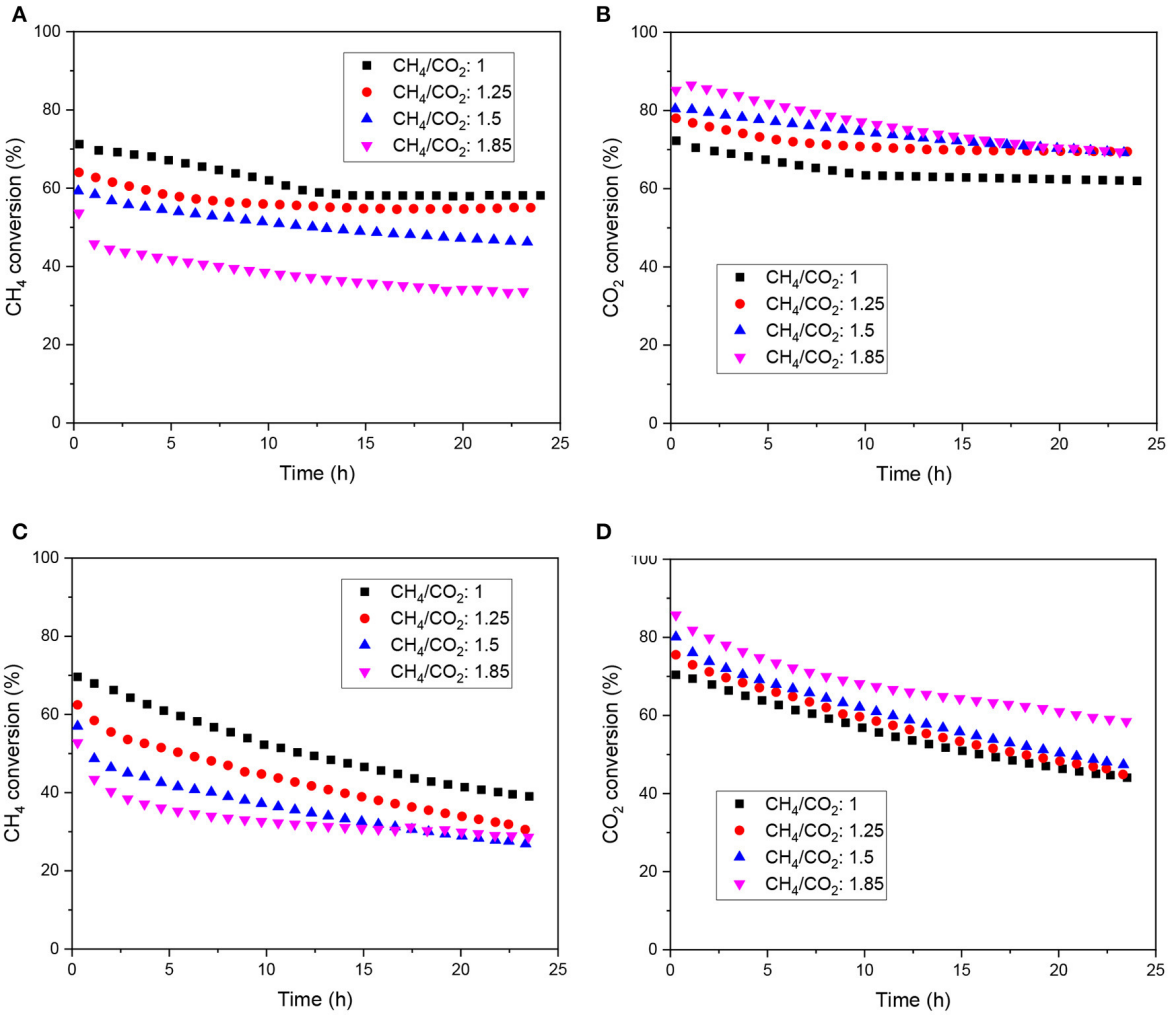
Stability test using different biogas feedstock on LNZ10 **(A)** CH_4_ conversion, **(B)** CO_2_ conversion and on Ni/LZ **(C)** CH_4_ conversion, **(D)** CO_2_ conversion.

Long-term stability tests were carried out at 700°C on the doped and supported catalysts, where carbon deposition is favored for each ratio according to Figure 1. CH_4_ and CO_2_ conversion as a function of time are displayed in Figure 4 for various biogas feedstocks. The doped catalyst (LNZ10) displayed relatively stable activity for biogas mixtures with relatively low methane content (CH_4_/CO_2_ = 1 and 1.25), however, a small deactivation was observed when using higher CH_4_/CO_2_ ratios. In particular, a deactivation of about 25% in conversion was observed when the reaction was performed under a biogas mixture of CH_4_/CO_2_ = 1.85, where carbon deposition is favored, owed to the excess of methane in the inlet stream.

On the other hand, the supported catalyst showed continuous deactivation for all the range of biogas feedstocks. A deactivation of about 30% in conversion was observed for the lowest methane containing biogas.

**Figure 5:**
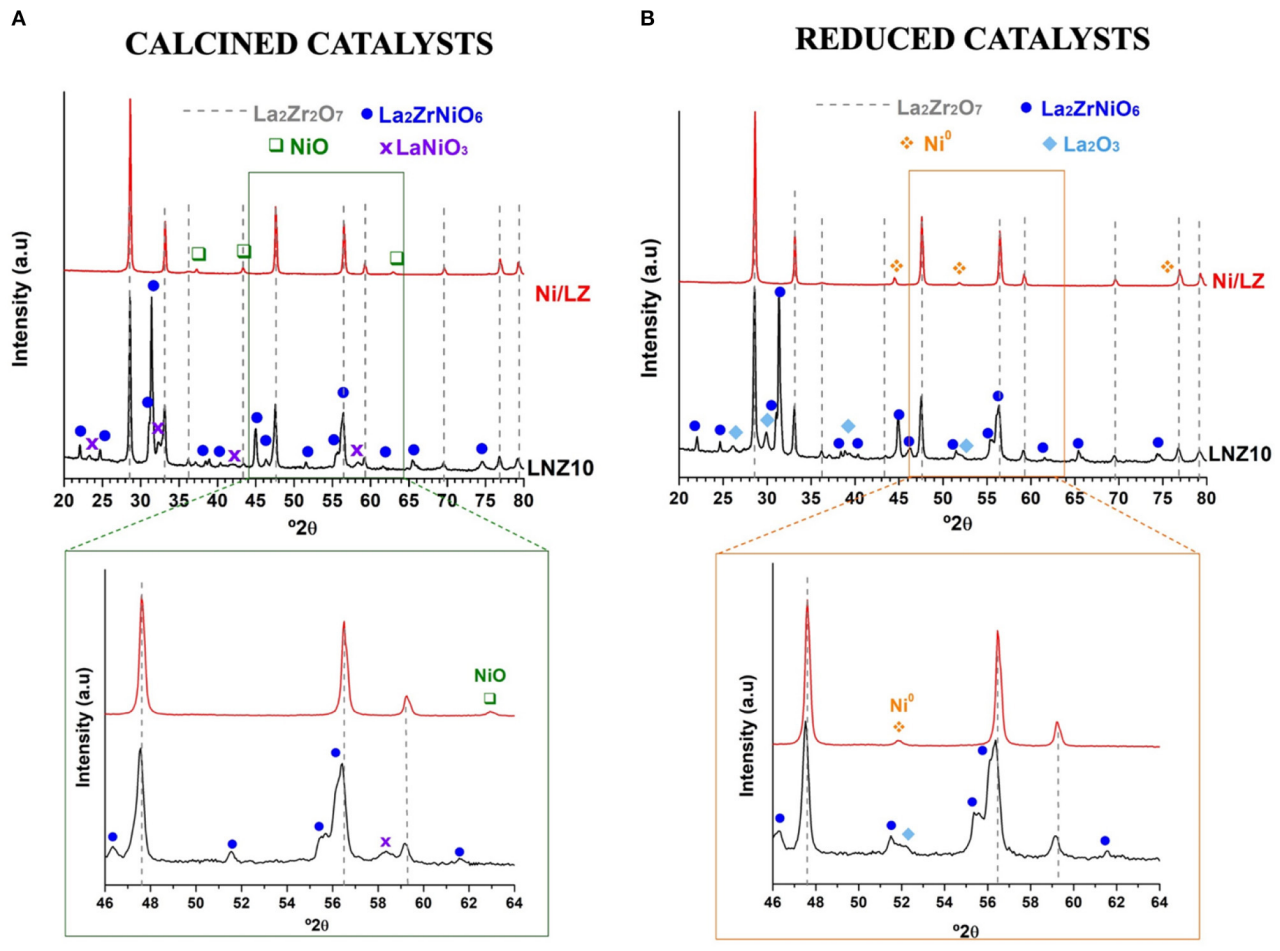
XRD of the **(A)** calcined and **(B)** reduced catalysts. Inset sections indicate the NiO and Ni° peaks analyzed for Scherrer crystallite size calculations.

The different catalytic behavior of both samples can be explained by the different Ni species in both catalysts. For one side, the calcined doped sample (LNZ10) presents the characteristic diffraction peaks of the La_2_Zr_2_O_7_ pyrochlore phase and the La_2_NiZrO_6_ rhombohedral double perovskite oxide phase (Figure 5) (le Saché et al., 2018, 2020). The presence of the latter, implies that the Ni% utilized in the synthesis method, surpassed the maximum substitution limit of the pyrochlore structure leading to the formation of this additional phase (Haynes et al., 2017). Zr and La seemed to be fully incorporated into the pyrochlore and/or the perovskite oxide phase, since no ZrO_2_ or La_2_O_3_ oxides are observed, however, traces of LaNiO_3_ are detected at 32.3°2θ in the calcined sample. Likewise, Ni seems to be fully incorporated into the mixed structures as no NiO is observed.

Regarding the calcined impregnated sample (Ni/LZ) diffraction peaks of NiO additionally to the La_2_Zr_2_O_7_ pyrochlore phase are evidenced. In this catalysts, the rhombohedral double perovskite oxide phase (La_2_NiZrO_6_) was not formed owed to the calcination process. In this sample, NiO crystallite size calculated by Scherrer Equation using the peak at 62,8°2θ was: 24 nm. When reduced, the impregnated sample displays the same XRD pattern, however, metallic Ni^0^ is evidenced at 44,5 and 51,8°2θ. The doped sample on the other hand, shows traces of La_2_O_3_ in addition to Ni^0^, resulting from the reduction of LaNiO_3_. In the reduced samples, no difference in the Ni^0^ crystallite size is evidenced. Ni^0^ crystallite size calculated by Scherrer Equation with the 44,5°2θ peak, was found to be 28 nm for both samples.

Results show that excess methane relative to the proportion of oxidant implies carbon formation, mostly owed to the cracking of methane (equation 5). As described by Luisetto et al. (2015), large Ni clusters seem to favor carbon deposition. Hence, the design strategy of the pyrochlore-perovskite clearly prevents carbon formation at relatively low CH_4_/CO_2_ ratio.

**Figure 6:**
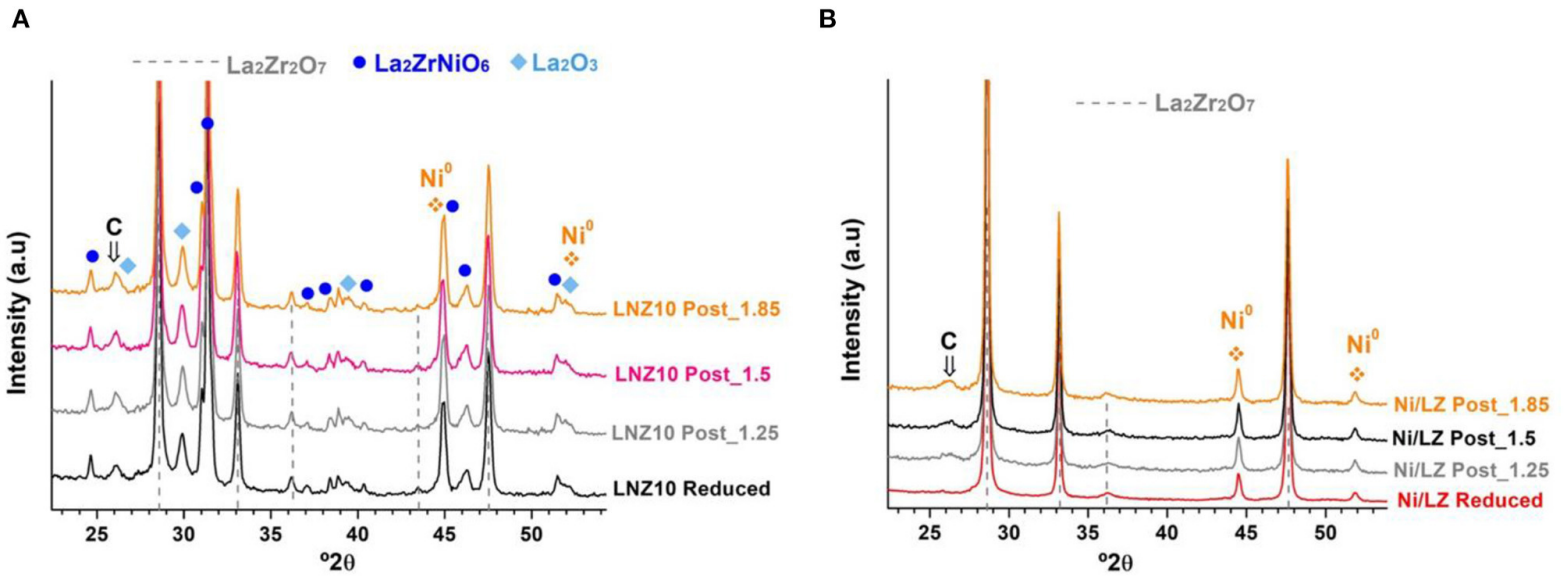
XRD of the post reacted samples under CH_4_/CO_2_ ratios higher than 1. **(A)** Doped sample and **(B)** supported sample.

**Figure 7:**
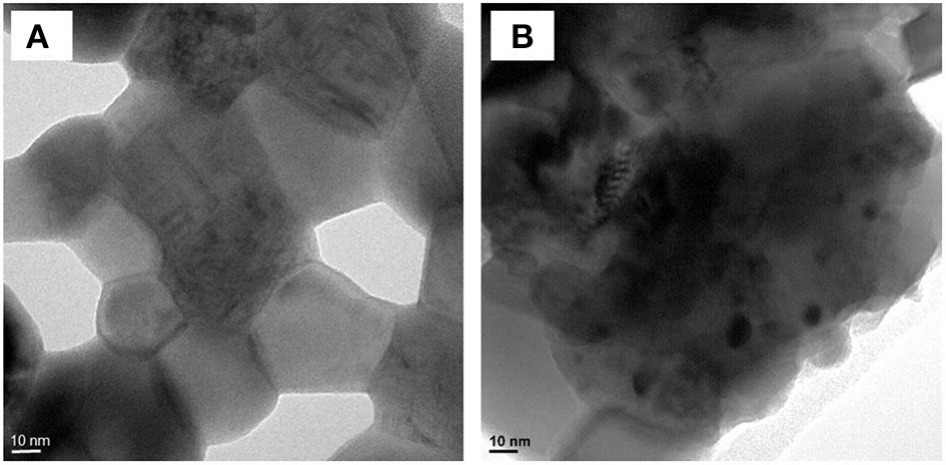
TEM of the doped sample **(A)** calcined and **(B)** post stability test under CH_4_/CO_2_: 1.

XRD of the post-reacted samples are shown in Figure 6. Besides the already described pyrochlore, perovskite and metallic Ni peaks, a peak corresponding to the graphite lattice plane (002) of carbon nanotubes appears around 26°2θ (Ferlauto et al., 2006). This peak increases with the CH_4_/CO_2_ ratio, especially on the supported sample, implying a higher carbon deposition with larger amounts of methane in the model biogas, which agrees with the deactivation observed in Figure 4 and the thermodynamic calculations. However, little Ni^0^ sintering was evidenced. Scherrer analysis of the peak at 44,4°2θ shows that the crystallite size varied between 26 and 29 nm for the doped sample and between 28 and 30 nm for the supported sample. The initial activity of the doped catalyst may be due to the Ni particles resulting from the reduction of LaNiO_3_. Later, the exsolution of Ni from the mixed-perovskite/pyrochlore phases may occur and provide new active sites (le Saché et al., 2018). Indeed, TEM images of the doped sample before and after reacting for 24 h under a CH_4_/CO_2_ ratio of 1 are shown in Figure 7. No Ni particle were distinguished on the calcined sample as it is integrated in various structures according to the XRD profile. After time dependent reaction, Ni particles can clearly be distinguished with diameters larger than 10 nm. These particles could result from the exsolution of Ni from the various phases and their subsequent sintering.

### Carbonaceous Deposits Evaluation

**Figure 8:**
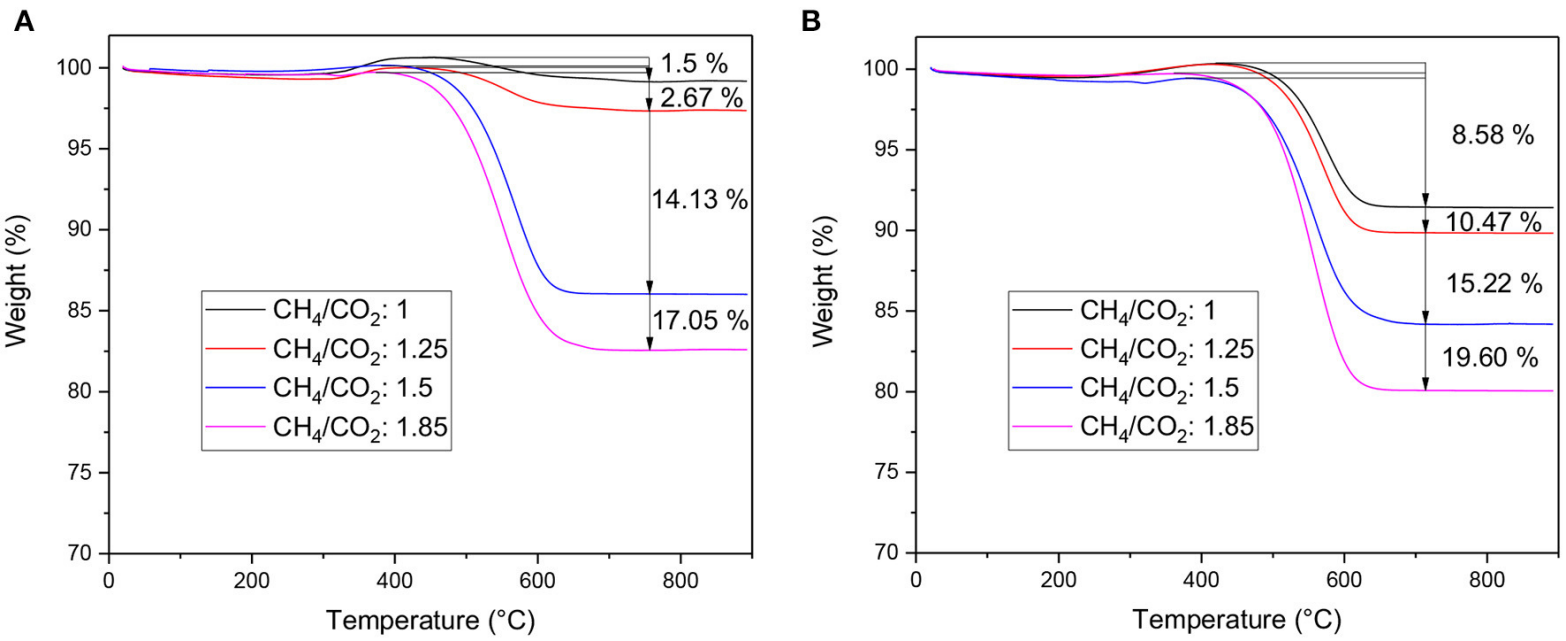
TGA of the samples after stability test. **(A)** LNZ10 and **(B)** Ni/LZ.

Thermogravimetric analysis was conducted on the samples after reacting for 24 h to estimate the carbon deposits formed during the reaction. Figure 8 shows the effect of CH_4_/CO_2_ ratio on the feed on coke deposition. A slight weight gain is observed on all samples at 400°C corresponding to the oxidation of nickel. Very limited carbon formation was detected on the doped catalyst after reforming of the model mixture and landfill waste produced biogas. This is in good agreement with the catalytic data. The activity stabilized after 15 h of reaction and showed limited deactivation. Only 15 mg_C_ ${\text{g}}_{\text{cat}}^{-1}$ was formed using model biogas whereas for the biogas mixture 2, 141 mg_C_ ${\text{g}}_{\text{cat}}^{-1}$ was formed. It appears that the doped catalyst is able to resist carbon deposition for CH_4_/CO_2_ mixture up to 1.25. Beyond this ratio, the catalyst displayed steady deactivation. In comparison, the impregnated catalyst displayed important amount of coke for all tested mixtures. An outstanding amount of coke was formed during the reforming of the model mixture, 85.8 mg_C_ ${\text{g}}_{\text{cat}}^{-1},$ despite the equimolar amount of oxidant. At higher CH_4_/CO_2_ ratios however, the amount of carbon deposits is quite similar between the two catalysts, highlighting the threshold of methane content that the doped catalyst can fully oxidize to CO.

## Conclusions

Herein we demonstrate the excellent activity of a Ni-substitute pyrochlore catalyst for biogas upgrading to syngas via dry-reforming processes. The stabilizing effect of the pyrochlore framework offers a unique environment for Ni active sites to be protected from severe deactivation resulting in highly dispersed Ni ensembles. Indeed, after the reaction, small Ni clusters are present on the surface of the catalyst as suggested by XRD. In fact, it is very likely that active Ni clusters are exsolved from the pyrochlore during DRM leading to highly dispersed Ni clusters which account for the high activity and stability of the catalyst during the reaction. For sake of fair comparison, a standard Ni supported on a pyrochlore structure was tested under the same conditions. The Ni-substitute pyrochlore systems outperform the behavior of the supported material for all the tested conditions. Especially during the reforming of methane-rich biogas mixtures, the small Ni particles allowed an exceptional carbon resistance in comparison to the reference impregnated catalyst. Beyond the structure robustness of this novel reforming catalysts, this work showcases its applicability to multiple biogas mixtures including surrogates of landfill, sewage, and organic wastes with different CH_4_/CO_2_ ratios. This is a very interesting result indicating that our Ni-doped pyrochlore catalysts is not active for DRM but it also offers flexibility in terms of biogas mixtures. We can actually fine-tune the end-product composition by implementing our catalyst to upgrade multiple biogas mixtures thus resulting in a unique flexible catalyst for bio-syngas production paving toward the way to sustainable chemical synthesis.

## Data Availability Statement

The raw data supporting the conclusions of this article will be made available by the authors, without undue reservation.

## Author Contributions

All authors listed have made a substantial, direct and intellectual contribution to the work, and approved it for publication.

## Conflict of Interest

The authors declare that the research was conducted in the absence of any commercial or financial relationships that could be construed as a potential conflict of interest.
